# A dataset of low-carbon energy transition index for Chinese cities 2003–2019

**DOI:** 10.1038/s41597-023-02815-7

**Published:** 2023-12-16

**Authors:** Yifan Shen, Xunpeng Shi, Zhibo Zhao, Jinghang Xu, Yongping Sun, Zhenliang Liao, Yingzhu Li, Yuli Shan

**Affiliations:** 1 School of Economics and Management, Tongji University, Shanghai, 200092 China; 2 Australia-China Relations Institute, University of Technology Sydney, Sydney, 2007 Australia; 3 School of Finance, Qilu University of Technology (Shandong Academy of Sciences), 58 Sangyuan Road, Jinan, 250100 China; 4 School of Geography, Earth and Environmental Sciences, University of Birmingham, Birmingham, B15 2TT UK; 5 Institute of State Governance, Huazhong University of Science and Technology, Wuhan, 430074 China; 6 School of Economics, Huazhong University of Science and Technology, Wuhan, 430074 China; 7 College of Environmental Science and Engineering, Tongji University, Shanghai, 200092 China; 8 School of Public Affairs, Zhejiang University, Hangzhou, 310058 China

**Keywords:** Energy justice, Sustainability, Climate-change mitigation

## Abstract

Cities are at the heart of climate change mitigation as they account for over 70% of global carbon emissions. However, cities vary in their energy systems and socioeconomic capacities to transition to renewable energy. To address this heterogeneity, this study proposes an Energy Transition Index (ETI) specifically designed for cities, and applies it to track the progress of energy transition in Chinese cities. The city-level ETI framework is based on the national ETI developed by the World Economic Forum (WEF) and comprises two sub-indexes: the Energy System Performance sub-index, which evaluates the current status of cities’ energy systems in terms of energy transition, and the Transition Readiness sub-index, which assesses their socioeconomic capacity for future energy transition. The initial version of the dataset includes ETI and its sub-indexes for 282 Chinese cities from 2003 to 2019, with annual updates planned. The spatiotemporal data provided by the dataset facilitates research into the energy transition roadmap for different cities, which can help China achieve its energy transition goals.

## Background & Summary

The energy sector, which accounts for two-thirds of global greenhouse gas emissions^1^, is a critical focus for climate policy. However, the transition from fossil fuels to renewable energy sources is a multifaceted process, necessitating significant technological and socioeconomic changes, as well as investment in infrastructure, market incentives, and public education^2–4^. The pace of this energy transition largely depends on the readiness of these supporting measures, which can vary across space and time. Therefore, it is essential to monitor the progress of energy transition at the sub-national level and identify the differentiated potential for future transitions to ensure fast and just transitions that do not leave vulnerable regions behind^5^.

Urban areas, which consume two-thirds of global energy and contribute to over 70% of global CO_2_ emissions^6^, play a key role in climate change mitigation. Achieving Sustainable Development Goal (SDG) 11, which aims for inclusive, safe, resilient, and sustainable cities^7^, necessitates a successful transition to low-carbon urban energy systems. Understanding the variations among cities and over time can aid in creating targeted and efficient energy transition roadmaps. China, the world’s leading carbon emitter with vast regional disparities, requires diversified efforts at subnational levels. Radical, one-size-fits-all actions, particularly if poorly timed, could result in unjust or unsustainable energy transitions^8^, potentially hindering the achievement of the ambitious “Double Carbon” climate goal of reaching a carbon peak by 2030 and carbon neutrality by 2060.

Tailored actions require a detailed profile of energy transition at the city level, which is yet to be generated for China. Firstly, the available literature on energy transition in Chinese cities is relatively limited and primarily focuses on energy consumption and associated carbon emissions, as exemplified in studies by Mi, *et al*.^9^, Shan, *et al*.^10,11^, and Yang, *et al*.^12^. Secondly, while energy transition is a complex issue requiring multi-level and multi-dimensional assessment, existing analytical frameworks often focus on specific aspects such as sustainability^13^, energy security^14^, and energy poverty^15^. Current studies on sub-national energy transition also exhibit similar biases, often focusing on a single dimension like transitional vulnerability^5^. Comprehensive frameworks to assess energy transition across countries have been proposed by the World Economic Forum (WEF)^16^ and World Energy Council (WEC)^17^, but these assessments have not been extended to the sub-national or city level. Therefore, this study aims to develop a city-level Energy Transition Index (ETI) to measure cities’ energy transition progress, with a particular focus on China.

Our city-level ETI framework, adapted from the national ETI proposed by the WEF^16^, has been modified to suit the city context in China. The city-level ETI comprises an energy system performance sub-index reflecting current status of cities’ energy systems in terms of energy transition, and a transition readiness sub-index evaluating their socioeconomic capacity for future energy transition. The energy system performance sub-index aggregates eight indicators, while the transition readiness sub-index comprises 25 indicators. The first version of the dataset presents the ETI of 282 Chinese cities from 2003 to 2019. This dataset, along with the two sub-indexes, will be updated annually. The 33 individual indicators used in the index composition are also available for transparency and verifiability.

The uniformly formatted spatiotemporal ETI data can provide robust support for further analysis of energy transition across different cities and between similar cities, leading to customized roadmaps for Chinese cities. The city-level ETI framework could also be informative for other developing economies, promoting faster energy transition and diversified measures at more detailed levels. Our conceptual framework and dataset are based on our previous peer-reviewed publication^18^. However, in Shen, *et al*.^18^, we only generated preliminary data for three years (2005, 2010, 2015). In this study, we extended the previous work and collected comprehensive data, resulting in a complete panel dataset covering 282 cities from 2003 to 2019 (17 years). This complete panel dataset can be utilized by many other studies that investigate the climate change and low-carbon energy transitions. The dataset developed in this paper can be downloaded freely from the website of International Society for Energy Transition Studies (ISETS, https://isets.org/data) and Figshre^19^. Our datasets serve as a comprehensive supplement to the national Energy Transition Index (ETI) provided by the WEF^16^, providing a more detailed measures of energy transition at the sub-national level. The dataset provides policymakers with a robust foundation for evidence-based decision-making, allowing them to design and implement targeted policies and strategies to accelerate energy transition in Chinese cities.

## Methods

**Fig. 1:**
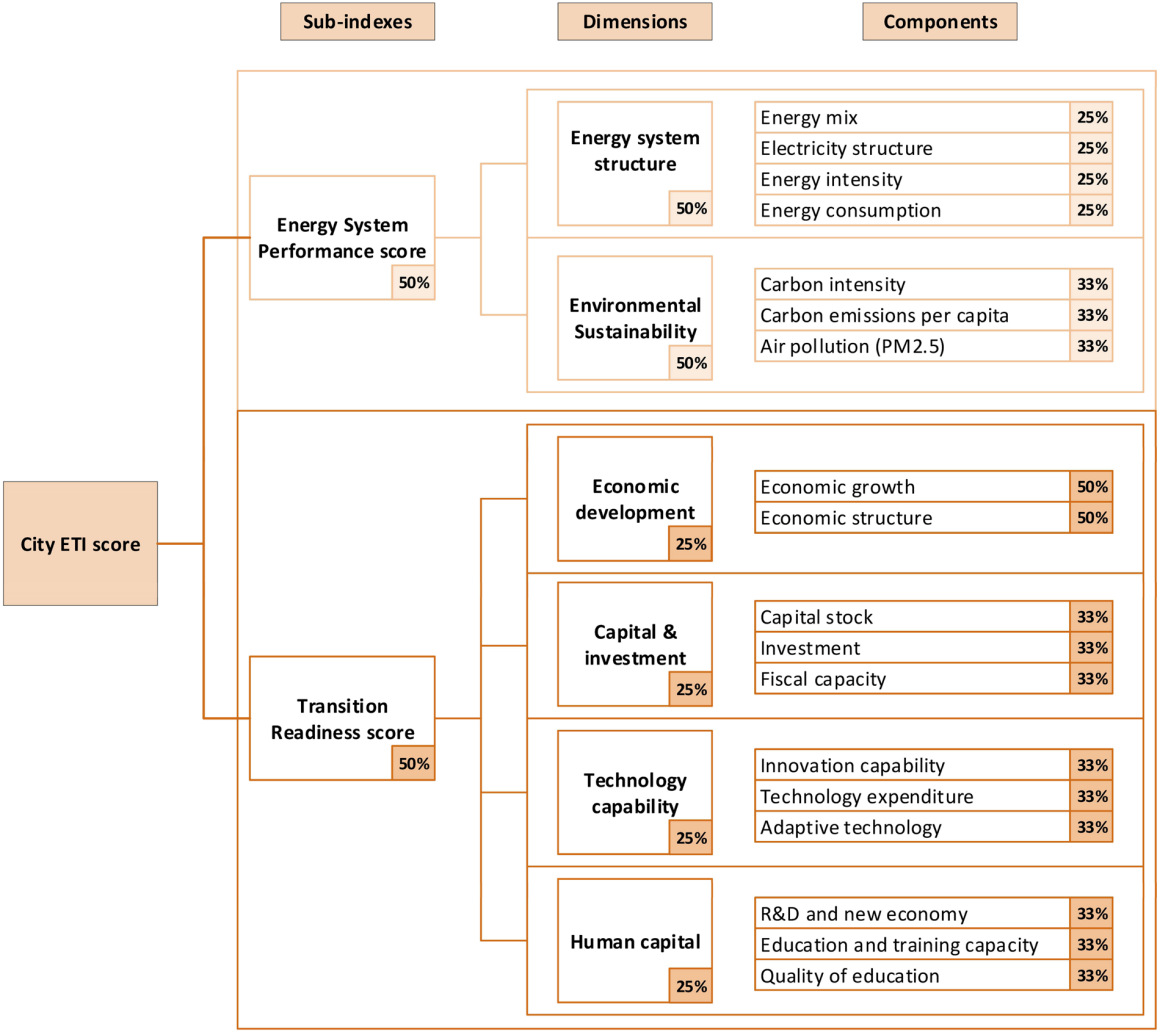
The conceptual framework of city-level energy transition index. Our city-level energy transition index (ETI) framework is adapted from the national ETI initially proposed by the World Economic Forum^16^. The adaptation involves a nuanced modification of the conceptual framework to suit the specific urban context. Each dimension is meticulously evaluated through critical components that bear relevance to the energy transition, guided by extant literature. More details, see the Methods section. Source: Our previous peer-reviewed publication^18^.

The conceptual framework of the city-level ETI is constructed by adapting WEF’s national ETI to a city context, and has been used in our previous peer-reviewed publication^18^. Following the WEF-ETI framework, the city-level ETI assesses energy transition from two sub-indexes: the energy system performance and the transition readiness. Each sub-index is measured from several dimensions, and each dimension includes several components. The index was created following the hierarchical structure, with equal weight assigned to indicators within each higher level. Figure 1 presents a diagram of our ETI framework. We have made several adjustments to the WEF-ETI conceptual framework to suit the city context and to account for the unique characteristics of energy transition in China. In particular, three WEF-ETI dimensions, namely “regulation and political commitment”, “institutions and governance”, and “energy access and security”, are no longer adopted. The first two were dropped because no heterogeneity exists in a centralized country, and the last one was irrelevant as China has achieved universal electricity access^20^. To better align with the naming of sub-indexes, the “economic development and growth” dimension and the “energy system structure” dimension were swapped. Generally, the “energy system structure” is more relevant to current energy system performance, and the economic indicators available at China’s city-level tend to reflect economic capability for future transition.

### The city-level ETI framework

Figure 1 presents our conceptual framework for assessing the measures of ETI at the city level. It consists of 2 sub-indexes, 6 dimensions, and 18 components. Each component is described using one or more indicators, and a total of 33 indicators have been collected. The sub-indexes exactly follow the WEF-ETI setting, while the dimensions were partially adapted for Chinese cities. For example, the “energy access and security” dimensions under the energy system performance sub-index was dropped, as the country has achieved nation-wide electricity access context. See Shen, *et al*.^18^ for more detailed discussions on the difference. Similarly, some indicators have been modified to better reflect energy transition in Chinese cities or to accommodate data availability (See Table 1 for detailed definition of each indicator).

**Table 1:** Further information on selected indicators.

Sub Index	Dimensions	Components	Indicators (unit)	Lower bound	Upper bound	Data sources
Energy system performance	Energy system structure	Energy mix	Share of coal in primary energy (%)	24.3%	95.2%	CEADs
		Electricity structure	Local coal consumption for power generation vs. total electricity consumption (kg standard coal equivalent/kWh)	0	2.09	CCSY, CEADs
		Energy intensity	Energy consumption per unit of GDP (standard coal equivalent /10^4^ yuan)	0.29	9.7	CCSY, CEADs
		Energy consumption	Energy consumption per capita (standard coal equivalent per capita)	0.41	13.6	CCSY, CEADs
			Electricity consumption per capita (kWh per capita)	488	16969	CCSY, CEADs
	Environmental sustainability	Carbon intensity	CO_2_ emissions per unit of GDP (t/10^4^ yuan)	0.55	5.76	CCSY, CEADs
		Carbon emissions per capita	CO_2_ emissions per capita within urban territory (t/per capita)	0.99	31.3	CCSY, CEADs
		Air pollution (PM2.5)	Annual average concentration of inhalable fine particulate matter (micrograms per cubic meter)	12.3	66.5	ACAG
Transition readiness	Economic development	Economic growth	Per capita GDP (yuan)	4597	151326	CCSY
			GDP growth rate (%)	3.5%	28.6%	CCSY
		Economic structure	Proportion of employment of mining employees in urban units at the end of the year (per 10^4^ capita)	0	533	CCSY
			Tertiary industry as percentage to GDP (%)	21.6%	57.6%	CCSY
	Capital & investment	Capital stock	Average annual balance of net fixed assets per capita (yuan per capita)	1048.5	84766.4	CCSY
			Proportion of urban construction land in the municipal area (%)	0.4%	34.1%	CCSY
			Per capita deposits of national banking system at the end of the year (yuan per capita)	4168.9	210182.4	CCSY
		Investment	Total investment in fixed assets per capita (yuan)	1624.7	80971	CCSY
			Amount of foreign capital per capita (US dollars per capita)	0.3	1007.8	CCSY
		Fiscal capacity	Public finance income per capita (yuan per capita)	167.9	14925.3	CCSY
	Technology capability	Innovation capability	China innovation and entrepreneurship index (0–100)	4.1	97.6	PKU-ORDP
			Proportion of subscribers of internet services (%)	1%	51%	CCSY
			Number of green invention and utility model patents applied for, per capita, in the year (number per 10^4^ capita)	0	2.04	CNRDS
		Technology expenditure	Expenditure for science and technology per capita (yuan per capita)	0.96	651.9	CCSY
		Adaptive technology	Ratio of industrial SO_2_ removed (%)	0.7%	87.4%	CCSY
			Ratio of wastewater centralized treated (%)	0.0%	97.9%	CCSY
			Ratio of consumption wastes treated (%)	0.0%	100.0%	CCSY
			Ratio of industrial solid wastes treated (%)	18.0%	100.0%	CCSY
	Human capital	R&D and new economy	Proportion of the persons engaged in scientific research, technical services and geological exploration industries (per 10^4^ capita)	2.2	98.2	CCSY
			Proportion of the persons employed in the information transmission, computer services and software industries (per 10^4^ capita)	2.6	67.3	CCSY
		Educational and training capacity	Proportion of employees in the education industry (per 10^4^ capita)	72.5	213.6	CCSY
			Number of full-time teachers in vocational secondary schools (per 10^4^ capita)	1.1	17.2	CCSY
			Number of full-time teachers in regular institutions of higher education (per 10^4^ capita)	0.4	49.7	CCSY
		Quality of education	Expenditure for education per capita (yuan per capita)	118.1	2831.3	CCSY
			Number of students enrolled in regular institutions of higher education (per 10^4^ capita)	5.9	886	CCSY

The energy system performance sub-index evaluates the current status of cities’ energy systems in terms of energy transition. It comprises two dimensions: energy system structure and environmental sustainability. Each dimension is evaluated by critical components related to the energy transition according to the literature. Within the energy system structure dimension, there are two components related to energy production side, namely energy mix and electricity structure, as well as two components pertaining to energy consumption side, namely energy intensity and energy consumption. Our city-level ETI framework aligns with the WEF-ETI framework for the energy system structure dimension, with the exception of energy demand growth, which is not considered as a snapshot of the energy transition status. The environmental sustainability dimension is defined similarly to the WEF-ETI framework and includes two components for carbon emissions (carbon intensity and carbon emissions per capita) as well as a component for air pollution, which is a significant negative consequence of fossil fuel consumption. See International Energy Agency^21^ for further information on energy transition measurements.

The transition readiness sub-index measures the preparedness of cities for future energy transition. This sub-index comprises four dimensions: economic development, capital & investment, technology capability, and human capital. Again, within each dimension are critical components related to the energy transition according to the existing literature. The economic development dimension gauges the macroeconomic preparedness to handle challenges related to the transition via two components: economic growth and economic structure. Economic growth examines whether the economy is undergoing rapid development, while economic structure examines the reliance on extractive and mining sectors^5,22–24^. For instance, cities with higher GDP levels possess greater resources and capacity to adapt to unfavourable changes from the energy transition. The other three dimensions are integral parts in production functions^25^, and can be adapted to measure the socioeconomic capability for energy transition. In particular, the capital and investment dimension examines the monetary resources available for supporting the transition. This includes the capital stock in the financial market, investment in the energy sector, and fiscal capacity of the government^26–28^. The technology capacity dimension measures both specific ready-to-use technology (adaptive technology), and economic resources (technology expenditure) and technology potential (innovation capacity) to develop new technologies^29–31^. The human capital dimension includes both human resources in R&D as well as new economy. Additionally, it evaluates education and training capacity that facilitates the transition of human capital, as well as the quality of education that determines the productivity of human resources^32–35^. Our comprehensive definition of transition readiness offers an advantage over the WEF-ETI framework at the city level. The literature suggests that while decarbonization is a crucial aspect, the energy transition also requires the support of infrastructure and investment; technologies and innovations; policy, institutions, and governance; and market incentives and public education^2,3^.

### Scope and data sources

This study selected 282 prefecture-level cities in China as the research sample based on data availability, and we calculated each city’s ETI scores from 2003 to 2019. The selected cities covered 98% of the country’s entire population, 99% of its gross domestic product (GDP), and 97% of its CO_2_ emissions in 2015^36^. Data for the indicators were collected from reputable open-access databases and official publications of the National Bureau of Statistics. Specifically, the energy and carbon emissions data were retrieved from Carbon Emission Accounts and Datasets for emerging economies (CEADs)^36–39^. The socioeconomic and environmental data mainly came from the China City Statistical Yearbook^40^ and the Atmospheric Composition Analysis Group^41^. The data about innovation capability were from the Peking University Open Research Data Platform (https://opendata.pku.edu.cn/file.xhtml?fileId=10543&version=4.1) and the Chinese Research Data Services Platform (https://www.cnrds.com/Home/Index#/). Table 1 provides detailed information on the indicators used in the city-level ETI framework.

### Calculation of city-level ETI scores

The city-level ETI scores were derived through standard composite index analysis, which has been widely used by international organizations in generating cross-entity comparable indexes, e.g., Human Development Index and SDG index by the United Nations, and national ETI scores by WEF. Particularly, the city-level ETI scores were calculated following two main steps.

#### Step 1: Normalization of indicator values

As indicators across different ETI dimensions vary dramatically in values, normalization that makes each indicator value range from 0 to 100 was conducted for comparability first. The general practice of min-max normalization was used following the two formulas below.

1 $$x{\prime} =\frac{x-\min (x)}{\max (x)-\min (x)}\times 100$$

2 $$x{\prime} =\frac{\max (x)-x}{\max (x)-\min (x)}\times 100$$

where *x* is the original value of each indicator, *x*′ is the normalized value, and max/min represents the maximum/minimum value of the indicator. Equation 1 is valid for indicators with higher values representing better performance (e.g., GDP per capita), and Eq. 2 is valid for indicators with lower values representing better performance (e.g., emission intensity). We used the common top and bottom 2.5th-percentile performers as the upper and lower bounds for all indicators in presenting baseline results. This approach has been employed in previous studies^42,43^ to minimize the potential impact of skewed data distributions on standardized values during normalization. As part of our robust analysis, we also experimented with alternative upper and lower bounds, such as the 1st and 99th percentile, and 5th and 95th percentile (For more details, see the Technical Validation section).

Normalized values exceeding the upper threshold were given a score of 100, whereas values below the lower threshold were assigned a score of 0. Values between the upper and lower bounds were distributed along a spectrum from the worst performance (score 0) to the best performance (score 100). An indicator with a score of 50 represents halfway towards achieving the best performance. These normalized scores allow assessing relative performance of energy transition over time and space. For example, a city that initially ranked lower than all other cities in a certain ETI indicator in both 2005 and 2015, but demonstrated improvement over time, would have a higher score for that ETI indicator in 2015 compared to 2005. However, its score would still be lower than that of the remaining cities in both years.

#### Step 2: Calculation of the ETI scores

The city ETI scores were calculated using arithmetic means in each step of aggregation, following the approach utilized by the WEF in generating national ETI^12^. As shown in Fig. 1, the ETI Index was computed by combining two sub-indexes with equal weight, and each sub-index was also obtained by assigning equal weight to the dimensions. In line with previous research^5,42,43^, there is no priori reason to give one sub-index or dimension greater weight than another. Within each ETI dimension, each component is equally weighted for the same reason. The Technical Validation section provides a discussion on the index’s reliability when employing different aggregation methods.

## Data Records

The dataset “City-level ETI scores” is made public under Figshare^19^. A total of 14,433 data records (Energy Transition Index, Energy System Performance Sub-index, and Transition Readiness Sub-index) are contained in the datasets. Of these,

- 4,794 are city-level Energy Transition Index (282 cities, from 2003 to 2019) [File tab ‘City’];
- 4,794 are city-level Energy System Performance Sub-index (282 cities, from 2003 to 2019) [File tab ‘City’];
- 4,794 are city-level Transition Readiness Sub-index (282 cities, from 2003 to 2019) [File tab ‘City’];
- 17 are national-level Energy Transition Index (from 2003 to 2019) [File tab ‘Nation’];
- 17 are national-level Energy System Performance Sub-index (from 2003 to 2019) [File tab ‘Nation’];
- 17 are national-level Transition Readiness Sub-index (from 2003 to 2019) [File tab ‘Nation’].

**Fig. 2:**
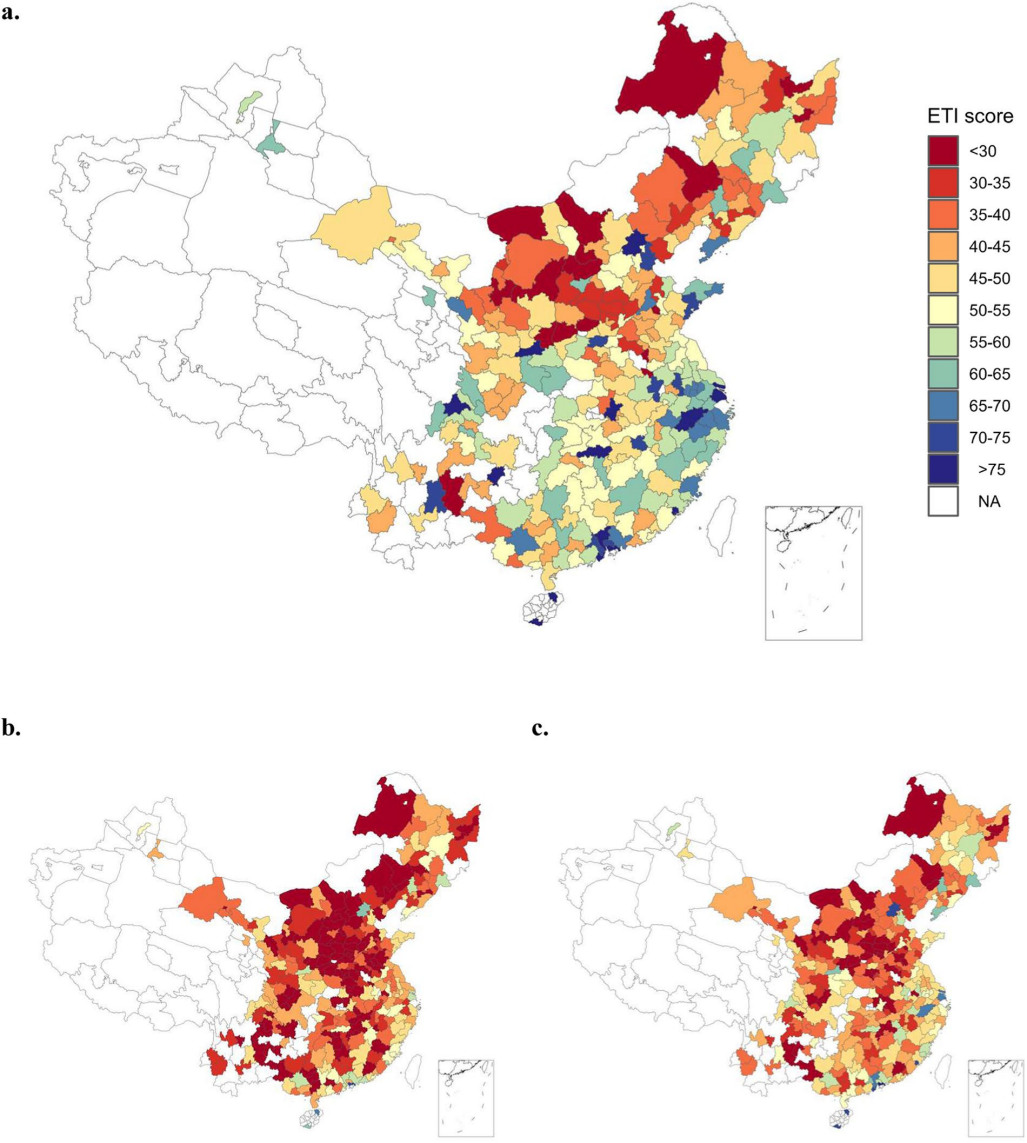
ETI scores for 282 Chinese cities. (**a**) 2019. (**b**) 2003. (**c**) 2010. More details on the generation process, see Methods. NA, data not available.

Our city-level ETI and its sub-indexes were constructed in a uniform format. As an example, Fig. 2 shows the spatial patterns 282 cities in energy transition by presenting the ETI scores in 2003, 2010, and 2019. The data illustrates the compelling trend that China has progressively enhanced its national average ETI score. However, it’s important to note that significant regional disparities persist within the country in relation to energy transition. This highlights the heterogeneity among cities and emphasizes the need for city-specific low-carbon roadmaps instead of relying on one-size-fits-all approaches.

## Technical Validation

### Sensitivity analysis for ETI scores

The robustness of the energy transition index (ETI) is examined by introducing uncertainties to some key assumptions. Specifically, the sensitivity test was performed over three alternative configurations, including: (1) adjusting upper and lower bounds; (2) successively excluding dimensions; and (3) exploring alternative aggregation methods. Overall, our findings demonstrate that the results generally remain robust when reasonable adjustments are made during the index construction. In particular, we experimented with alternative upper and lower bounds of 0.01 and 0.99, 0.05 and 0.95. We also experimented with the geometric mean instead of arithmetic mean when generating the ETI. In addition, we experimented with a successive exclusion of six dimensions in the ETI framework to check if the results are sensitive to one particular dimension.

**Fig. 3:**
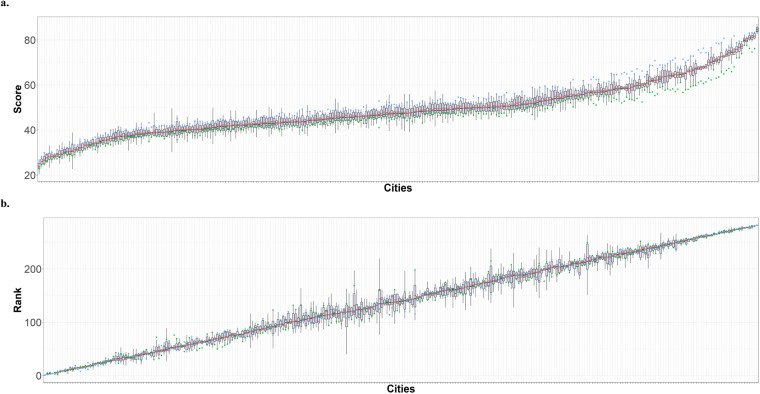
Sensitivity analysis for city-level ETI scores. (**a**) Sensitivity analysis results for scores. (**b**) Sensitivity analysis results for rankings. The robustness of the energy transition index (ETI) is scrutinized through varied upper and lower bounds, distinct aggregation methods, and a systematic exclusion of dimensions within the ETI framework. The cities are arranged in order of their medians, with the baseline ETI value highlighted in red. Results predicated on alternative upper and lower bounds of 0.01 and 0.99, as well as 0.05 and 0.95, are denoted in blue and green, respectively. In each box plot, the central rectangle delineates the interquartile range (IQR) from the first quartile (Q1) to the third quartile (Q3), wherein the line segment inside the rectangle represents the median. When the maximum observed ETI scores surpass Q3 + 1.5 × IQR, the upper whisker is Q3 + 1.5 × IQR. Otherwise, the upper whisker corresponds to the maximum observed ETI score. When the minimum observed ETI scores fall below Q1 − 1.5 × IQR, the lower whisker is Q1 − 1.5 × IQR. Otherwise, the lower whisker aligns with the minimum observed ETI score. Analogous principles apply for the ETI rankings.

The resulting variation of cities’ scores and rankings are depicted in Fig. 3. The cities are ordered according to their median. The baseline value of the ETI is marked in red. The results based on alternative upper and lower bounds of 0.01 and 0.99, 0.05 and 0.95 are marked in blue and green, respectively. In each box plot, the central rectangle spans the first quartile Q1 to the third quartile Q3, which is the interquartile range (IQR) (IQR = Q3 to Q1), while the line segment inside the rectangle shows the median. The upper whisker is set at Q3 + 1.5 × IQR when the maximum observed ETI scores exceed this value. Conversely, the lower whisker is set at Q1 - 1.5 × IQR if the minimum observed ETI scores fall below Q1 - 1.5 × IQR. In other cases, the upper and lower whiskers take the values of the maximum and minimum observed ETI scores, respectively. Figure 3 indicates that the ranking of cities remains robust for those in the top and bottom quintiles of the scale. While the cities falling in between exhibit a somewhat wider interquartile range, the original ETI ranking remains close to the median. More than half of the cities shifted by only a maximum of one position from the median rank. Major differences in scores and ranks are usually witnessed when a dimension that represents a city’s comparative advantage or weakness in transition is excluded. However, testing alternative results by excluding dimensions showed that both ETI rankings and scores are not likely to be systematically driven by the outlier in any single dimension of the energy transition.

### Comparison with national index from the WEF

**Fig. 4:**
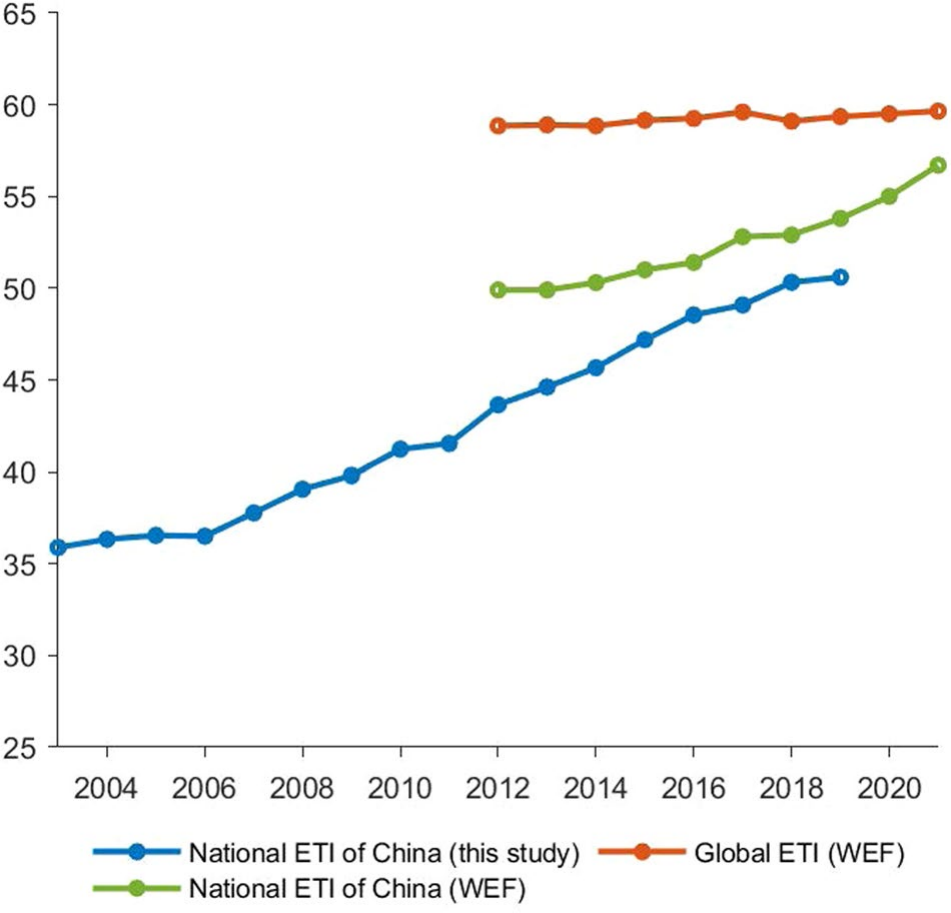
Comparison with national index from the WEF. The blue line is the aggregated national ETI of China generated in this study, which is based on the average of ETI scores across 282 cities. The green and red lines are China’s and global ETI scores reported by the WEF, respectively.

To verify the validity of our constructed index, we also compared our national ETI index aggregated by the energy transition performance of 282 cities with the national energy transition index provided by WEF^16^, which benchmarks the state of the energy transition in 115 countries (including China) from 2012 to 2021. As indicators are not completely the same, the two indexes are expected to have similar trends during the overlapped period but differ in absolute level. The comparison in Fig. 4 confirms the expectation: both indexes show that China has performed well in energy transition in recent years. Figure 4 also presents the aggregated WEF global energy transition index, which indicates that progress in energy transition is not a common phenomenon. In fact, China is one of the countries that have best performance in energy transition in the past decade, together with India and Sub-Saharan African nations according to the WEF^16^.

### Capturing the stylized facts in cities’ energy transition

Figure 2 has shown the geographic distribution of ETI scores across cities, which captures the stylized facts in cities’ energy transition. The cities with lowest ETI scores are clustered in Northeast and Central China, particularly Shanxi and Inner Mongolia. These regions are the country’s main areas for energy (especially coal) production. The heavy dependence on coal and the ordinary performance in socio-economic development make these cities face great challenges in the energy transition. In contrast, more socio-economically developed regions, such as Jing-Jin-Ji, Yangtze River Delta, and Pearl River Delta, have the highest ETI scores and above average energy system performance. The city-by-city analysis of ETI scores also has similar findings. For example, Ordos performed much better than other energy production cities, which is primarily due to its high level of transition readiness. This city’s GDP per capita is among the highest in China, which could support future energy transition with better socioeconomic capacity. This is also in line with the fact that Ordos recently became the center of low-carbon energy in China (For an overview of achievements and planning of the low-carbon industry in Ordos, see a recent report^44^).

**Fig. 5:**
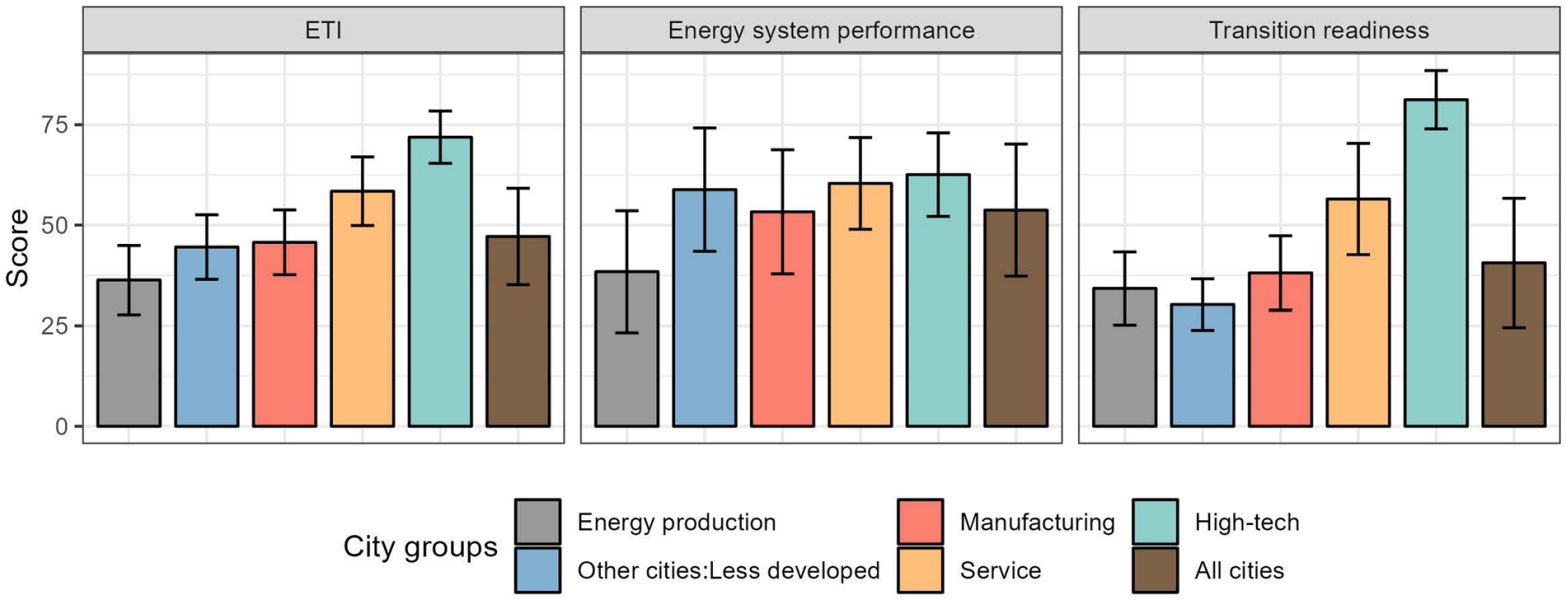
Stylized facts of ETI scores for different city groups. The bars illustrate the mean values of the variables, with vertical lines within each bar denoting the positive one standard deviation (+1 SD) of the respective variables.

To further validate our results, we categorized the 282 Chinese cities into five functional groups mainly based on their industrial output structure and GDP per capita. The groups include: energy production cities (n = 52), manufacturing cities (n = 93), service cities (n = 44), high-tech cities (n = 16), and other less developed cities (n = 77). Figure 5 illustrates the mean values and standard deviations (SDs) of ETI scores for each city group. The high-tech cities had the highest average ETI scores (71.9), while the energy production cities recorded the lowest average ETI scores (36.3) due to their lowest energy system performance and underperforming transition readiness. The service (58.4), manufacturing (45.7), and other cities (44.5) lay between them. While the energy production cities recorded a significantly lower energy system performance compared to the others, it is the difference in transition readiness that drives the group heterogeneity. The service cities and high-tech cities had significantly higher transition readiness scores than the other three types of cities. In summary, these results could accurately depict the state of energy transition among various city groups. As such, our datasets serve as a comprehensive supplement to the national Energy Transition Index (ETI) provided by the World Economic Forum (WEF), providing a more detailed measures of energy transition at the sub-national level.

### Limitations

Our dataset has several limitations, but we will work on these limitations in the future to measure and monitor the energy transition in Chinese cities more accurately.

1. Our dataset includes 282 cities, representing more than 95% of China’s population, GDP, and carbon emissions. However, there are other cities in the country for which data required for the Energy Transition Index (ETI) composition are partially unavailable. This may be due to a lack of attention to energy-related issues or socio-economic limitations affecting data collection. It is important to recognize that these cities also require growing concern over energy transition. Future research should aim to include these cities to ensure a more comprehensive assessment of energy transition across China.
2. Subject to data availability, again, the evaluation in this study failed to explicitly measure the renewable energy industry or its development potential in each city. Yang, *et al*.^12^ have made pioneering efforts in estimating urban renewable energy production at the city scale, which can serve as a valuable reference for future research. Future application of our index system could also possibly provide more accurate assessment of city-level energy transition by incorporating dimensions and indicators such as cumulative solar photovoltaic and wind capacity, renewable power generation, and jobs created in the renewable energy industry.

## Data Availability

Code generated by our analysis, including the *R* scripts for data preparation and composite index analysis, is available on GitHub at https://github.com/shentroy/Code_city_ETI.
